# Case report: Therapeutic response of front-line cadonilimab plus chemotherapy on patient with advanced lung adenocarcinoma harboring STK11 genetic aberration

**DOI:** 10.3389/fimmu.2024.1485358

**Published:** 2024-12-09

**Authors:** Du Feng, Huixin Jiang, Gengjia Chen, Wenhui Guan, Lin Yi, Yue Zhu, Yijia Li, Gengda Huang, Bin He, Junlong Tang, Yujie Tang, Jiyuan Zeng, Wensheng Zhou, Jiayu Shi, Zhanhong Xie, Ming Liu, Xiaohong Xie, Xinqing Lin, Chengzhi Zhou

**Affiliations:** ^1^ Department of Respiratory and Critical Care Medicine, State Key Laboratory of Respiratory Disease, National Clinical Research Center for Respiratory Disease, National Center for Respiratory Medicine, Guangzhou Institute of Respiratory Health, the First Affiliated Hospital of Guangzhou Medical University, Guangzhou, Guangdong, China; ^2^ Nanshan School, Guangzhou Medical University, Guangzhou, Guangdong, China; ^3^ The First School of Clinical Medicine, Guangzhou Medical University, Guangzhou, Guangdong, China; ^4^ Department of Stomatology, the First Affiliated Hospital of Guangzhou Medical University, Guangzhou, Guangdong, China

**Keywords:** STK11 mutation, lung adenocarcinoma, case report, cadonilimab, front-line therapy

## Abstract

The STK11 gene mutation is a common genetic alteration in non-small cell lung cancer (NSCLC) and is significantly associated with poor responses to current immunotherapy regimens. Despite its prevalence, there is currently no established standard for front-line treatment in this subtype of NSCLC, underscoring the increasing need for personalized therapeutic strategies. In this report, we present a case of a patient with STK11-mutant NSCLC who was treated with first-line cadonilimab (10mg/kg) in combination with pemetrexed (500mg/m^2) plus carboplatin (AUC=5), resulting in a notable extension of progression-free survival (PFS). This case highlights the potential efficacy and feasibility of combining immunotherapy with chemotherapy in patients with STK11-mutant NSCLC. Additionally, we provide a review of recent advancements in research related to STK11 mutations in lung cancer as reported in the literature.

## 1 Introduction

Lung cancer remains one of the most prevalent malignancies worldwide, with high rates of morbidity and mortality. Among these cases, non-small cell lung cancer (NSCLC) accounts for approximately 80% of all lung cancers (1). In recent years, Immune checkpoint inhibitors (ICI) have revolutionized the treatment landscape for lung cancer, offering new therapeutic options. However, the efficacy of immunotherapy can be compromised by specific genetic mutations, such as those in the serine/threonine kinase (STK11)/LKB1 gene, which is one of the most frequently inactivated tumor suppressors in NSCLC (2).

The STK11 gene plays a crucial role in cellular damage repair processes and is closely associated with reduced levels of tumor-infiltrating lymphocytes. Located at chromosome 19p13.3, the gene comprises 12 exons and encodes a protein that is distributed within the nucleus, cytoplasm, and mitochondria (2). Mutations in STK11/LKB1 impair its ability to activate the downstream adenosine monophosphate-activated protein kinase (AMPK) signaling pathway, which, in turn, disrupts the inhibition of the mammalian target of rapamycin (mTOR) pathway, thereby promoting tumor grow and proliferation (3). In lung adenocarcinoma (LUAD), irrespective of the kirsten rat sarcoma viral oncogene homolog (KRAS) status, alterations in STK11/LKB1 are closely linked to a “cold” immune microenvironment, resulting in a diminished response to ICIs, such as programmed death-1 (PD-1) inhibitors (4, 5).

Despite the involvement of STK11 mutations in numerous clinical trials, a standardized front-line treatment specifically for NSCLC patients with STK11 mutations remains elusive (6). The Phase III POSEIDON trial demonstrated that first-line treatment with a combination of durvalumab, tremelimumab, and chemotherapy significantly improved overall survival (OS) compared to chemotherapy alone, across both STK11/KEAP1/KRAS mutant and wild-type NSCLC subgroups. This suggests that combining immunotherapy with chemotherapy holds potential for patients with STK11 mutations (7, 8).

Cadonilimab is a bispecific IgG-single-chain Fv fragment (ScFv) antibody that simultaneously targets PD-1 and cytotoxic T-Lymphocyte-associated protein 4 (CTLA-4) (9). It has shown a favorable safety profile in both first-line and second-line treatments for advanced lung cancer, with relatively few adverse reactions reported (9–12). A Phase II single-arm clinical trial investigating the efficacy of Cadonilimab in NSCLC demonstrated that while it was safe, its effectiveness as a second-line treatment was limited, and no subgroup analysis based on specific mutation types was conducted. Thus, the role of Cadonilimab as a first-line treatment for NSCLC patients with STK11 mutations remains to be clarified.

Given these insights, we present a case of an NSCLC patient with an STK11 mutation who achieved a durable partial response (PR) after receiving Cadonilimab combined with chemotherapy as a front-line treatment. This case highlights the potential utility of Cadonilimab in treating NSCLC patients with this challenging genetic profile.

## 2 Case presentation

The patient, a 60-year-old male, presented to our hospital with a two-week history of cough and a recent lung cancer diagnosis identified during a routine check-up one week prior. Laboratory tests revealed significantly elevated level of lung cancer tumor markers, including carcinoembryonic antigen (CEA) at 156.00 ng/mL, carbohydrate antigen 125 (CA 125) at 238.00 U/mL, carbohydrate antigen 153 (CA 153) at 64.60 U/mL, cytokeratin-19 fragment (CYFRA 21-1) at 5.05 ng/mL, and squamous cell carcinoma antigen (SCC) at 1.80 ng/mL. Chest and abdominal CT scan showed an occupying lesion in the right upper lung, suggestive of peripheral lung cancer, as well as pathological compression fractures in the T9, T11, and L3 vertebrae.

**Figure 1:**
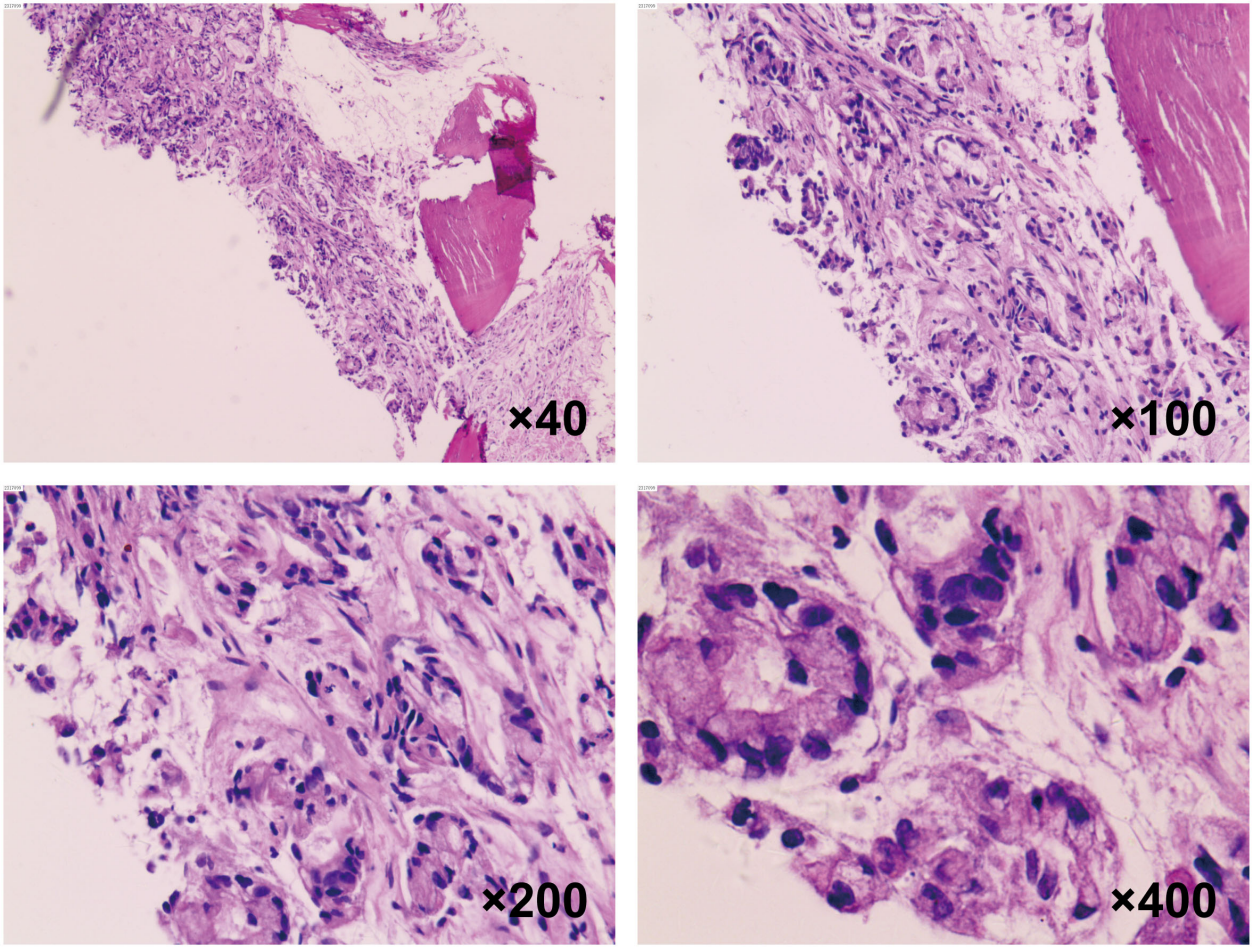
Histopathological analysis of vertebral metastasis biopsy. H&E staining of the spinal metastatic lesion biopsy at 40×, 100×, 200× and 400× magnification, demonstrating the cellular characteristics of the lung adenocarcinoma.

To confirm the diagnosis, a biopsy of the L3 bone metastasis was performed, with the pathology report indicating metastatic adenocarcinoma infiltrating bone tissue (**Figure 1**). Immunohistochemistry results showed positivity for Cytokeratin7 and Villin, while markers such as Thyroid Transcription Factor-1, NapsinA, Cytokeratin20, Caudal Type Homeobox 2, Special AT-rich Sequence-Binding Protein 2, and Prostate-Specific Antigen were negative. Next-generation sequencing revealed mutations in STK11, along with KRAS and TP53 mutations (**Supplementary Material**). Based on these clinical findings, the patient was diagnosed with LUAD with bone metastasis (T1cN3M1, Stage IVB), with an Eastern Cooperative Oncology Group performance status score of 1. However, PD-1 and PD-L1 expression levels were not assessed for this patient.

**Figure 2:**
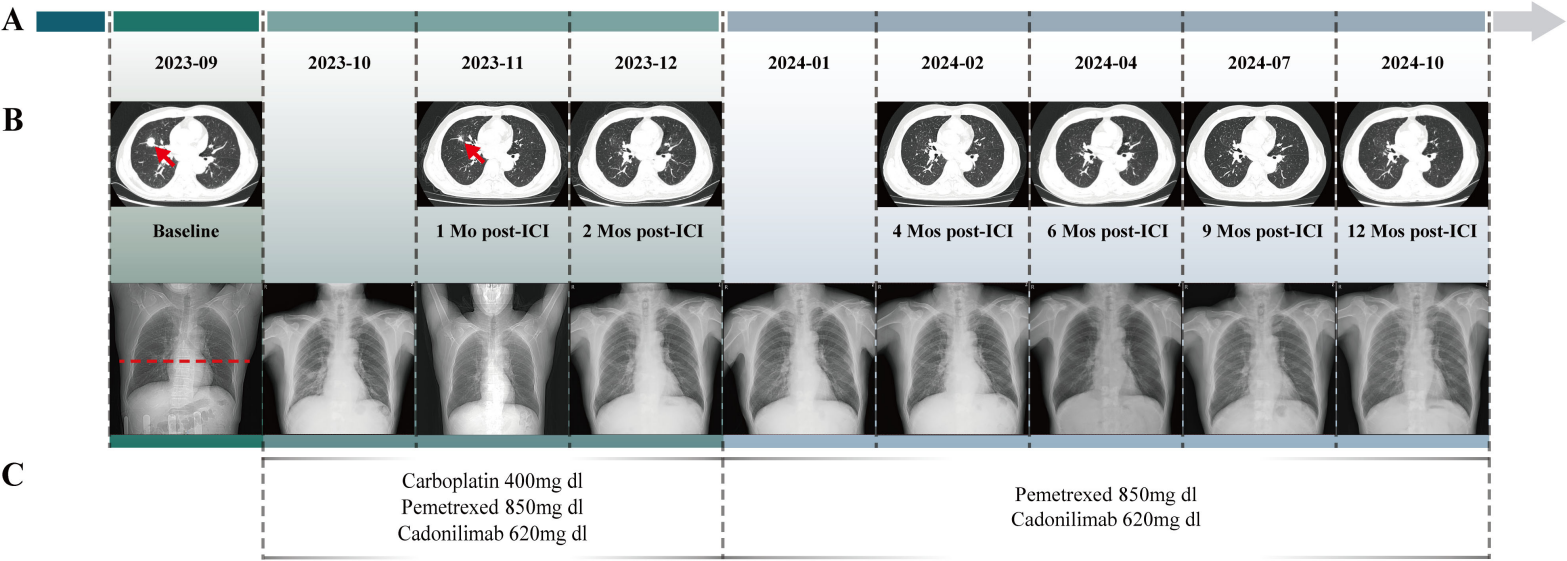
The Clinical Course of Lung Adenocarcinoma. **(A)** Timeline showing the clinical course. **(B)** Change of the tumor lesion revealed by radiological examinations (CT scans and chest X-rays). Radiological examinations demonstrated the progression of the lung adenocarcinoma. The baseline tumor on September 11, 2023, is marked by a red arrow, with its corresponding level on chest X-ray indicated by red dashed lines. The subsequent imaging on November 19, 2023 demonstrated a partial response with significant tumor shrinkage. Serial surveillance scans were performed, with the latest imaging obtained on October 21, 2024. **(C)** Timeline of the patient’s systemic treatment course. From October 8 to December 31, 2023, chemotherapy commenced with Pemetrexed and Carboplatin, along with the immunotherapy agent Cadonilimab. Since January 22, 2024, subsequent chemotherapy cycles with Pemetrexed and Cadonilimab were administered.

The timeline begins with the baseline assessment in September 2023, followed by monthly intervals after ICI treatment, extending through a comprehensive 12-month evaluation (**Figures 2A, B**). The patient received chemotherapy with pemetrexed (0.85g) and carboplatin (400mg), combined with Cadonilimab (620mg) immunotherapy, administered on October 8, October 28, and November 18, 2023; and December 10, and December 31, 2023 (**Figure 2C**). For consistent monitoring, we selected and maintained the imaging plane that demonstrated the largest cross-sectional area of the primary lesion at initial diagnosis. After two cycles of treatment, a follow-up chest CT on November 19, 2023, revealed a 68% reduction in the lung lesion size, achieving a PR as per the Response Evaluation Criteria in Solid Tumors version 1.1. Follow-up chest X-ray examinations from September 2023 to October 2024 demonstrated complete resolution of pulmonary inflammation with restoration of normal lung markings (**Figure 2B**). The patient then continued with consolidation therapy consisting of pemetrexed (0.85g) and Cadonilimab (620mg) every three weeks. By August 2024, follow-up evaluations showed that tumor marker levels had returned to normal: CEA at 1.92 ng/ml, CA125 at 12.20 U/mol, CA153 at 25.60 U/mol, and CYFRA 21-1 at 2.64 ng/mL. Throughout the treatment period, the patient did not experience any significant adverse events, such as hepatotoxicity, immune-related pneumonitis, musculoskeletal pain, or rash. On October 21, 2024, chest CT scans demonstrated further reduction in lesion size (**Figure 2B**).

## 3 Discussion

In this study, we present a unique case of a patient with non-small cell lung cancer (NSCLC) harboring an STK11 mutation who achieved a sustained PR for 1 year with first-line treatment using Cadonilimab (a bispecific antibody targeting both PD-1 and CTLA4) combined with chemotherapy (administered every three weeks). This outcome is noteworthy, as durable responses in the patient with STK11 mutation is rarely observed with conventional immunotherapy. However, it is important to acknowledge that the link between STK11 mutation and immunotherapy efficacy remains incompletely understood. To date, there is no definitive evidence elucidating how STK11 mutations influence response to immune checkpoint inhibitors, nor are there well-established guidelines for the optimal first-line treatment strategy for NSCLC patients with this mutation profile. Further studies are needed to explore the underlying mechanisms and to establish standardized therapeutic approaches for this challenging subset of patients.

Conflicting evidence exists regarding the efficacy of immune checkpoint inhibitors in patients with NSCLC harboring STK11/LKB1 mutations. On the one hand, several studies suggest that these patients may derive benefit from a combination of immunotherapy and chemotherapy. For instance, exploratory analyses from two Phase III trials, presented at the American Association for Cancer Research 2020, indicated that pembrolizumab, whether used as monotherapy or in combination with chemotherapy, was associated with improved outcomes in patients with STK11-mutated NSCLC (13, 14). Additionally, another Phase III trial demonstrated that nivolumab combined with chemotherapy as first-line therapy achieved significantly better overall survival (OS) in metastatic NSCLC with STK11 mutations, with a median OS of 20.7 months compared to 9.5 months with chemotherapy alone (15). The Phase III POSEIDON trial further supported these findings, showing that a combination of dual ICI therapy (Durvalumab and Tremelimumab) plus chemotherapy led to a 3-year OS rate of 25.8% in patients with STK11 mutations, compared to only 4.5% for chemotherapy alone (16). However, it is crucial to acknowledge that these findings are not universally accepted. Critics argue that most clinical trials were not specifically designed to address the impact of STK11 mutations on ICI efficacy, leading to limited sample size and thus, inconclusive results. For instance, a subgroup analysis showed no statistically significant difference in OS when comparing atezolizumab monotherapy with docetaxel in patients with STK11 mutations (HR = 0.669; 95%CI: 0.380–1.179; P = 0.669) (17). Wang et al. have also reported that STK11-mutated patients do not derive significant benefits from ICIs (18). A recent meta-analysis by Xu’s team, which included 14 retrospective studies with 4,317 patients, found that the objective response rate (ORR) for STK11-mutated patients treated with immunotherapy was only 10.1% (19). Importantly, many of these studies utilized immunotherapy as a later-line treatment, which may have contributed to the low response rates. Given this heterogeneity in study results, there is still no consensus on the optimal treatment regimen for NSCLC patients with STK11 mutations. Based on current evidence, however, we hypothesize that moving immunotherapy to the first line, utilizing a dual ICI approach, and combining it with chemotherapy may enhance outcomes for these patients. The case presented here serves to validate this hypothesis to some extent. It is the first reported instance of an STK11-mutated NSCLC patient achieving a durable PR of 1 year with first-line treatment using a PD-1/CTLA-4 bispecific antibody in combination with chemotherapy. Our finding suggests that a dual immune blockade strategy, when initiated early, may be beneficial even in patients with historically poor responses to conventional ICIs.

Recent findings have demonstrated that tumors with STK11 mutations exhibit an immunosuppressive microenvironment characterized by the accumulation of myeloid cells and a deficiency in cytotoxic CD8+ T cells. The study highlights that CTLA-4 blockade can remodel this immunosuppressive TME by increasing the infiltration of effector CD4+ T cells, particularly Th1 cells, and by enhancing the presence of antigen-presenting cells, such as MHCII+ macrophages and iNOS-expressing dendritic cells. This immune reprogramming may help restore anti-tumor immunity even in tumors harboring STK11 mutations (20–22). Furthermore, preclinical models suggest that this dual blockade approach, showing that the combination of PD-1 and CTLA-4 inhibitors has a synergistic effect that goes beyond the additive benefits of each used alone. The synergy is achieved by reducing the proportion of exhausted phenotype cytotoxic CD8+ T cells and enhancing active effector T cell populations (23). The clinical efficacy of dual blockade as a first-line treatment of NSCLC has been corroborated by multiple clinical trials and meta-analyses, demonstrating that adding a CTLA4 inhibitor to PD-1/PD-L1 inhibitors significantly improves patients in terms of ORR, OS, and PFS (24–27). Lastly, the chemotherapy component in our regimen likely contributed to the patient’s positive response. Chemotherapy has been shown to potentiate anti-tumor immunity by inducing immunogenic cell death and enhancing the presentation of tumor antigens, thereby boosting the effectiveness of concurrent immunotherapy (28). The combination of these therapies may have collectively contributed to the durable PR observed in the patient, suggesting that a comprehensive, multimodal approach could be essential for treating NSCLC patients with STK11 mutations.

While it remains challenging to pinpoint the exact genomic alterations responsible for our patient’s exceptional therapeutic response, this case underscores the complexity and potential research value of individual genomic landscapes in influencing treatment outcomes. The presence of co-mutations further complicates the clinical picture and potentially affects the therapy’s efficacy (29). In this case, the patient harbored mutations in multiple key genes, including KRAS, TP53, and STK11. It is well established that TP53 mutations can promote tumor growth by increasing tolerance to a higher mutational burden; however, they also lead to a greater neoantigen load, which can enhance immunogenicity. Moreover, the loss of p53 function has been linked to the activation of the nuclear factor κB (NF-κB) pathway. Consequently, KRAS-mutant tumors bearing co-mutations with TP53 have shown improved clinical response to PD-1 axis immunotherapy, with significantly better PFS and OS (5). However, despite these findings, the majority of existing studies indicate that these co-mutations often correlate with poorer treatment efficacy and prognosis. Specifically, KRAS and STK11 co-mutations are associated with distinct metabolic phenotypes and a complex tumor microenvironment, suggesting that STK11 inactivation in KRAS-mutant lung adenocarcinoma serves as a major driver of primary resistance to PD-1 axis blockade, as confirmed by multiple clinical trials (5, 30). Although we are unable to precisely identify the specific genetic alterations that contributed to the patient’s remarkable response with minimal adverse effects, this case provides insights into potential personalized treatment strategies for further investigation (31).

Our study has several limitations that warrant discussion. First, while PD-1 expression levels are considered one of the most robust clinical predictors of response to anti-PD-1/PD-L1 therapies, we were unable to directly assess PD-1 expression in our patient. This is a significant limitation because patients with higher TMB often exhibit lower PD-L1 scores but can still respond favorably to PD-1 blockade. The absence of PD-1 expression data makes its challenging to determine whether the observed therapeutic efficacy is primarily due to Cadonilimab or influenced by intrinsic PD-1 expression levels (18). To address this, future studies should include more comprehensive profiling to disentangle the contributions of Cadonilimab from individual variations in PD-1 levels. Second, the functional phenotype of the STK11 mutation in our patient remains unclear. Given that STK11 mutations are associated with heterogeneous immune microenvironments and varying responses to immunotherapy, the lack of functional characterization limits our understanding of how this mutation may have impacted the patient’s immune response. This uncertainty underscores the need for well-designed preclinical studies and randomized clinical trials to elucidate the interplay between STK11 mutations, PD-1, and CTLA-4 pathways, ultimately guiding the development of standardized treatment protocols. Additionally, a limitation in our imaging analysis should be noted. The CT images presented throughout the treatment period were limited to a single consistent plane from the initial diagnostic scan. While this consistent imaging plane allows for a standardized comparison over time, it may not fully capture the dynamic changes in tumor volume and morphology that could occur in other regions. This could potentially lead to an underestimation or misinterpretation of the actual therapeutic response. Future studies should consider incorporating more comprehensive imaging techniques, such as volumetric assessments, to provide a more accurate evaluation of treatment effects.

## 4 Conclusion

In conclusion, Cadonilimab plus chemotherapy as a first-line treatment for a patient with stage IVB lung adenocarcinoma harboring the STK11 mutation demonstrated significant clinical efficacy while maintaining an excellent safety profile, with no severe adverse events observed. These findings suggest that early, comprehensive immunotherapy combined with chemotherapy may provide substantial advantages for patients with advanced NSCLC who harbor STK11 mutations. Specifically, the front-line regimen of Cadonilimab alongside pemetrexed and carboplatin shows promise as an effective therapeutic strategy in this challenging patient population, potentially paving the way for more personalized treatment approaches that optimize outcomes while minimizing toxicity. Further investigation in large clinical cohorts will be essential to confirm these results and establish this combination as a standard of care.

## Data Availability

The original contributions presented in the study are included in the article/**Supplementary Material**. Further inquiries can be directed to the corresponding authors.
