# Supplementary material for: Case report: Therapeutic response of front-line cadonilimab plus chemotherapy on patient with advanced lung adenocarcinoma harboring STK11 genetic aberration
Source: Front Immunol. 2024 Dec 9;15:1485358. doi: 10.3389/fimmu.2024.1485358 (PMC11663897; doi:10.3389/fimmu.2024.1485358)
Supplement: Supplementary file 1 [file DataSheet1.pdf]

肿瘤组织样本基因突变检测报告单

1. 送样信息

姓名：	年龄：	取样部位：转移灶
性别：男	样本编号：	检测申请日期：2023-09-14
送检科室：骨脊柱外科（沿江）	住院号/门诊号：	样本接收日期：2023-09-20
送检医生：	病理号：	报告日期：2023-09-27
样本类型：石蜡包埋组织+血液对照		
临床诊断：转移性腺癌		

2. 检测结果

具有临床意义的检测结果（I/II 类变异）

基因组变异	突变频率/拷贝数/胚系突变	变异等级	FDA/NMPA 已批准本癌种的药物（敏感性，证据等级）	FDA/NMPA 已批准其他癌种的药物（敏感性，证据等级）
KRAS<br>NM_033360.2<br>p.G12C<br>EX2	21.5%	I 类	Adagrasib(敏感，A 级)<br>Sotorasib(敏感，A 级)<br>阿法替尼(耐药，C 级)<br>阿来替尼(耐药，C 级)<br>克唑替尼(耐药，C 级)<br>厄洛替尼(耐药，C 级)<br>吉非替尼(耐药，C 级)<br>埃克替尼(耐药，C 级)<br>奥希替尼(耐药，C 级)<br>阿美替尼(耐药，D 级)	Binimetinib(敏感，C 级)<br>依维莫司(耐药，D 级)<br>西罗莫司(耐药，D 级)<br>替西罗莫司(耐药，D 级)
TP53<br>NM_000546.5<br>p.C176S<br>EX5	23.5%	II 类	-	-
TP53<br>NM_000546.5<br>p.G154V<br>EX5	5.7%	II 类	-	-
VHL<br>NM_000551.3	1.4	II 类	-	Belzutifan(敏感，C 级)

缺失				
CHEK2<br>NM_007194.3<br>缺失	1.4	II 类	-	尼拉帕利(敏感，C 级)<br>奥拉帕利(敏感，C 级)<br>Rucaparib(敏感，C 级)<br>Talazoparib(敏感，C 级)
BRCA1<br>NM_007294.3<br>缺失	1.4	II 类	-	氟唑帕利(敏感，C 级)<br>尼拉帕利(敏感，C 级)<br>奥拉帕利(敏感，C 级)<br>帕米帕利(敏感，C 级)<br>Rucaparib(敏感，C 级)<br>Talazoparib(敏感，C 级)<br>奥拉帕利联合贝伐珠单抗(敏感，C 级)
STK11<br>NM_000455.4<br>缺失	1.2	II 类	-	依维莫司(敏感，D 级)<br>西罗莫司(敏感，D 级)<br>替西罗莫司(敏感，D 级)
RAD51C<br>NM_058216.1<br>缺失	1.2	II 类	-	尼拉帕利(敏感，C 级)<br>奥拉帕利(敏感，C 级)<br>Rucaparib(敏感，C 级)<br>Talazoparib(敏感，C 级)

临床意义不明变异（III 类变异）

基因	检测结果	功能区域	变异类型	突变频率/拷贝数
TGFBR2	NM_001024847.2<br>p.E315K (c.943G>A)	EX5	错义突变	22.1%
LRP1B	NM_018557.2<br>p.G4278C (c.12832G>T)	EX84	错义突变	13.7%
GNAS	NM_000516.4	all exon	扩增	4.2
KEAP1	NM_203500.1	all exon	缺失	1.0

胚系变异检测结果

基因	转录本	碱基改变	氨基酸改变	基因型	变异意义
-	-	-	-	-	-

检测者:　　　　**审核者**:　　　　**报告日期：**2023-09-27
